# Multi-Objective Process Parameter Optimization of Ultrasonic Rolling Combining Machine Learning and Non-Dominated Sorting Genetic Algorithm-II

**DOI:** 10.3390/ma17112723

**Published:** 2024-06-03

**Authors:** Junying Chen, Tao Yang, Shiqi Chen, Qingshan Jiang, Yi Li, Xiuyu Chen, Zhilong Xu

**Affiliations:** College of Marine Equipment and Mechanical Engineering, Jimei University, Xiamen 361000, China; chenjunying@jmu.edu.cn (J.C.); yangtaoyinaifan@163.com (T.Y.); chensq_17@163.com (S.C.); robinly0316@163.com (Y.L.); jdcxy@126.com (X.C.); zhilong.xu@163.com (Z.X.)

**Keywords:** machine learning, multi-objective optimization, ultrasonic rolling, surface integrity

## Abstract

Ultrasonic rolling is an effective technique for enhancing surface integrity, and surface integrity is closely related to fatigue performance. The process parameters of ultrasonic rolling critically affect the improvement of surface integrity. This study proposes an optimization method for process parameters by combining machine learning (ML) with the NSGA-II. Five ML models were trained to establish relationships between process parameters and surface residual stress, hardness, and surface roughness by incorporating feature augmentation and physical information. The best-performing model was selected and integrated with NSGA-II for multi-objective optimization. Ultrasonic rolling tests based on a uniform design were performed, and a dataset was established. The objective was to maximize surface residual stress and hardness while minimizing surface roughness. For test specimens with an initial surface roughness of 0.54 µm, the optimized process parameters were a static pressure of 900 N, a spindle speed of 75 rpm, a feed rate of 0.19 mm/r, and rolling once. Using optimized parameters, the surface residual stress reached −920.60 MPa, surface hardness achieved 958.23 HV, surface roughness reduced to 0.32 µm, and contact fatigue life extended to 3.02 × 10^7^ cycles, representing a 52.5% improvement compared to untreated specimens and an even more significant improvement over without parameter optimization.

## 1. Introduction

The fatigue performance of key components is crucial for ensuring long-term, reliable service of high-end equipment. The surface integrity of these components directly affects their fatigue performance. Surface integrity characteristics include the surface roughness, the component microstructure, the microhardness distribution, the residual stress distribution, and surface features (such as surface defects and macroscopic cracks), among others [1,2]. Components with lower surface roughness values also have lower localized surface stress concentrations, which delays crack initiation [3,4]. Components with higher surface hardness values also have higher yield stresses in their surface layers, which inhibits crack initiation and growth [5,6]. Many studies have demonstrated that higher surface residual compressive stresses inhibit microscopic crack growth [7,8]. Because the initiation and growth of microscopic cracks eventually lead to fatigue fracture failure [9,10], the fatigue lives of key components can be effectively enhanced by reducing the surface roughness and increasing the surface hardness and residual compressive stress values, i.e., improving the surface integrity [11,12].

The ultrasonic rolling method uses high-frequency vibration of a rolling ball to induce intense plastic deformation in the surface layer of a metallic material; this process generates surface residual compressive stresses and produces surface hardening while simultaneously decreasing the surface roughness. Therefore, it is widely used to improve component surface integrity [13,14,15]. Ultrasonic rolling is a multifactorial coupling process; the surface roughness, surface hardness, and residual stress are all affected by the process parameters, such as the ultrasonic rolling static pressure, the spindle speed, the feed rate, and the number of rolls, which together form a complex nonlinear problem. Liu et al. [16] indicated that the use of improper process parameters can produce poor surface integrity strengthening effects and even produce micro-cracks and defects on the test bar surface. Many physical specimens are needed when performing trial-and-error experiments to obtain the best surface integrity; thus, a cumbersome parameter optimization process is necessitated that involves a substantial experimental workload and a long research and development cycle. Establishing a connection between the process parameters and the surface integrity characteristics can effectively improve the process parameter optimization efficiency. Zheng et al. [17] constructed an analytical model for ultrasonic rolling to relate static pressure, amplitude, and residual stresses. Zhang et al. [18] constructed a model for the residual stresses that was based on the rolling pressure, rolling ball radius, and test bar radius; however, some other parameters were not comprehensively considered. Jiao et al. [19] proposed an ultrasonic rolling contact area coefficient and constructed a planar ultrasonic rolling model based on the static pressure and this contact area coefficient. In summary, there is currently no reliable mathematical model that relates the ultrasonic rolling process parameters to the surface integrity improvement effect. The lack of efficient means to select process parameters for achieving optimal surface integrity, thereby enhancing component fatigue performance, is a pressing issue in the engineering application of ultrasonic rolling.

In recent years, data science (as represented by data-driven ML) has become a popular tool used in scientific research and technology development. It is widely used to analyze and predict the characteristics of complex engineering processes [20]. Feng et al. [21] developed a digital twin-driven intelligent gear health management method for assessing the process of gear surface degradation. Motta et al. [22] developed a model based on the random forest (RF) and Gaussian process (GP) algorithms to predict surface roughness during cylindrical turning of workpieces. Kryzhanivskyy et al. [23] constructed an ML model to predict the surface integrity of a component using the cutting parameters. Li et al. [24] proposed a data-driven ensemble learning model to predict surface roughness during additive manufacturing and validated the proposed model using collected temperature and vibration signals. Their results indicated that the proposed method could predict surface roughness with high accuracy. Chang et al. [25] used a data-driven ML model to relate laser melting process parameters to the quality of a finished part. To quickly select the process parameters for laser melting of 316L-Cu multi-material parts with compositional gradients, Rankouhi et al. [26] developed an ML model based on a multivariate GP to predict the density and surface roughness of a finished part with given laser melting process parameters. Yu et al. [27] developed a physics-informed neural network to accurately predict the shrinkage defect volume and the number of defects in titanium alloy shells based on process parameters. The authors further proposed that the predictive model could be used to optimize process parameters. Mambuscay et al. [28] employed a random forest model to directly predict Vickers hardness values from scanned indentation images, eliminating the need for diagonal measurements. Liu et al. [29] constructed a VMD-GRU model to predict the deformation of deep excavations, with experimental results confirming the model’s accuracy and effectiveness. If the processing results can be predicted based on process parameters, the machine learning model will effectively assist in optimizing process parameters.

Improving surface integrity through ultrasonic rolling involves evaluating surface roughness, surface hardness, and residual stress. It is inevitable that conflicting optimization objectives for the best improvement effect will need to be faced, such as causing deterioration of surface roughness while improving residual stress and surface hardness [30]. The NSGA-II algorithm can effectively address complex problems involving multiple conflicting optimization objectives [31,32]. Deng et al. [33] successfully designed a new composition for a nickel-based high-temperature alloy using NSGA-II and obtained excellent validation through experiments. Dong et al. [34] simultaneously optimized the mechanical strength and resistivity of graphite nanoparticle-reinforced cement-based composite materials using NSGA-II, obtaining suitable design parameters. Yang et al. [35] used NSGA-II to optimize the process parameters of laser–magnetic hybrid welding. The effectiveness of the process parameters was confirmed through confirmation experiments. NSGA-II has been widely applied in optimizing the process parameters of multiple machining performances [36,37,38]. Currently, there is no research on applying NSGA-II to optimize ultrasonic rolling process parameters.

Although previous studies have made some progress in establishing the relationship between process parameters and residual stress, the developed models do not provide clear optimal process parameters. Moreover, current non-data-driven research methods have not yet established the relationship between process parameters, hardness, and surface roughness. This paper proposes a process parameter optimization method by combining a machine learning model with the NSGA-II multi-objective evolutionary algorithm, addressing the gaps in the existing research on ultrasonic rolling. To be specific, this paper proposes a method for training ML models based on physical information and feature augmentation to enhance their predictive accuracy. Relationships between process parameters and surface residual stress, hardness, and surface roughness are established. Five ML models’ predictive accuracy is compared, and the best-performing model is selected. NSGA-II is then used to search for the optimal process parameters based on the best-performing ML model, and the effectiveness of the optimal process parameters is discussed. In the engineering practice of ultrasonic rolling, this method allows for the rapid selection of appropriate process parameters based on different requirements for residual stress, surface hardness, and surface roughness. This significantly improves the efficiency of selecting process parameters for various optimization goals, providing reliable guidance for applying ultrasonic rolling in engineering practice. The main innovation and contribution of this paper can be concluded as follows: (1) Reconstructing input features based on feature enhancement and constructing the maximum contact stress during the ultrasonic rolling process as a physical information feature. The ML model is trained by combining feature enhancement with physical information features. (2) Developing a hybrid multi-objective optimization method by integrating ML and NSGA-II, which is applied for the first time to optimize the quality of surface integrity improvement in ultrasonic rolling, including surface residual stress, hardness, and surface roughness.

## 2. Methodology

**Figure 1:**
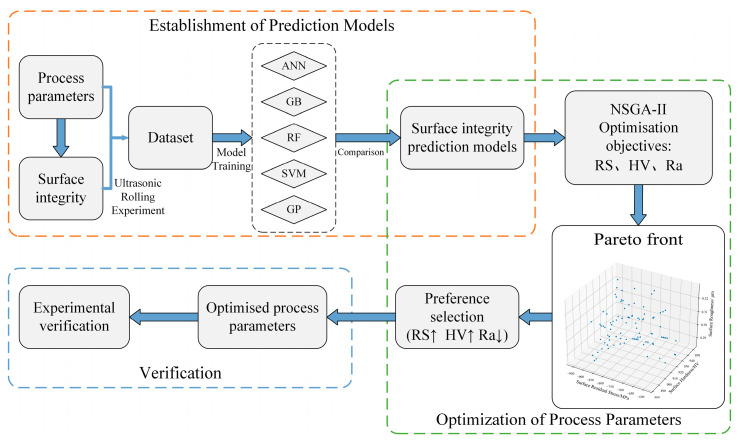
ML combined with NSGA-II process parameter optimization flow.

In this paper, ML and NSGA-II were adopted to optimize the parameters of the ultrasonic rolling process to achieve the best improvement in surface integrity. Figure 1 illustrates the overall process. The ultrasonic rolling test was carried out to collect process parameters and surface integrity data. A training dataset for the ML model was established. The prediction performances of ANN, GB, RF, SVM, and GP were compared [39], and the best model was selected as the objective function of NSGA-II. The optimization goal was to maximize residual stress and surface hardness while reducing surface roughness. The optimal process parameters were effectively searched by NSGA-II, which were then verified through ultrasonic rolling experiments to ensure their validity.

### 2.1. Surface Integrity Prediction Model

#### 2.1.1. Dataset Establishment

The key component material investigated during this study was 20CrNiMo steel (8720 steel in the AISI and SAE standards). 20CrNiMo steel is a high-quality, low-carbon alloy with good machinability and plasticity characteristics. Because it is a low-carbon steel, it also has excellent toughness and impact resistance characteristics. It is generally used after it has undergone carburizing and quenching processes, which are a series of heat treatment processes used to improve surface properties. However, under cyclic-loading service conditions, the fatigue lives of key components composed of carburized and quenched 20CrNiMo steel are still below the expected values. During this study, a standard contact fatigue test bar made from carburized and quenched 20CrNiMo steel was taken as the research object, and ultrasonic rolling was used to enhance the surface integrity and thereby also improve the fatigue performance.

**Figure 2:**
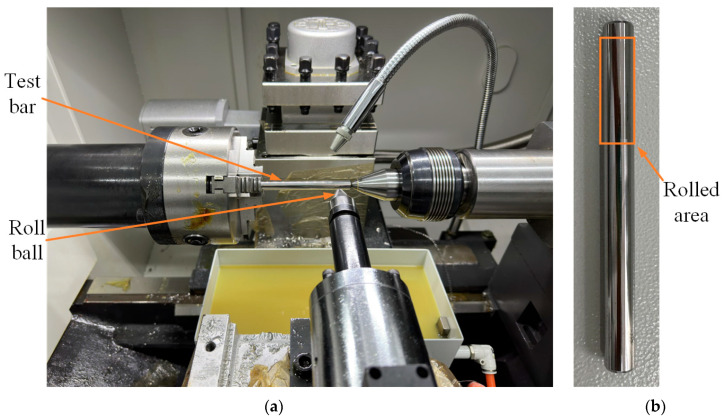
Ultrasonic rolling equipment and 20CrNiMo alloy samples: (**a**) ultrasonic rolling equipment, (**b**) 20CrNiMo alloy samples.

High-quality experimental data are the basis for data-driven ML model training. The ultrasonic rolling process is influenced by multiple synergistic factors. The experimental design involves multiple factors with various levels, and at the same time, the experimental workload must be reduced as much as possible. Therefore, a uniform design based on centered L_2_ discrepancy was used to design the experimental scheme during this study. Based on previous work conducted by the research group [40], the static pressure (P), spindle speed (S), feed speed (F), and number of rolls (R) were selected as the primary ultrasonic rolling influencing factors investigated with regard to surface integrity improvements. Six levels of values were chosen for each of the four factors; therefore, an experimental program that contained 24 groups of experiments was designed. The HKC30-50 ultrasonic rolling equipment (Shandong Huayun Electromechanical Technology, Jinan, China) used during the study is shown in Figure 2a. The surface roughness (Ra, µm) of the test bars was measured using the KEYENCE VK-X1000K device (Keyence, Kansas City, MO, USA) at a 100× magnification. The surface hardness was measured using a FALCON 500 Vickers hardness tester (Innovatest, Maastricht, The Netherlands) with a load of 100 g. The surface residual stress was measured with an HDS-I X-ray stress tester using the inclination method. The test bars had dimensions of 110mm×ϕ10mm, as shown in Figure 2b.

The experimental results of different process parameters based on the experimental scheme are shown in Table 1. The table also recorded the initial surface roughness of each test bar. We randomly selected 21 samples as the training dataset and the remaining 3 samples as the test dataset. Table 2 shows the descriptive statistics of train and test data split. We calculated the mean, standard deviation (sted), minimum, first quartile (Q1), median, third quartile (Q3), maximum, interquartile range (IQR), skewness, and kurtosis, and performed a two-sample Kolmogorov–Smirnov (KS) test [41]. The KS test results indicate that the significance value for the residual stress dataset is 0.63, the hardness dataset is 0.39, and the surface roughness dataset is 0.99. All values are greater than the significance level of 0.05, indicating that the distributions of the training and test datasets are the same.

#### 2.1.2. Feature Engineering

**Figure 3:**
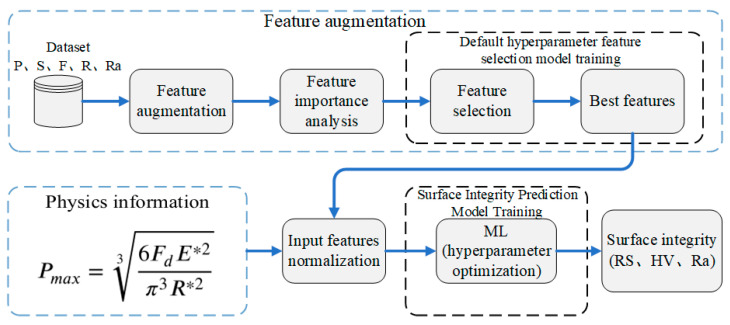
Process for establishing a surface integrity prediction model based on feature augmentation and physical information features.

In the specific practice of machine learning models, the size of the sample affects the prediction accuracy of the machine learning model. Therefore, a certain amount of data is still needed to train the model, which cannot truly meet the demand for reducing the experimental workload in engineering practice. Under the condition of limited samples, it is still necessary to study methods to improve the prediction accuracy of machine learning models. Dai et al. [42] constructed high-dimensional features from the original primary features, selected effective features from the high-dimensional features to improve the original dataset, and ultimately improved the prediction capability of the ML model. Bai et al. [43] used the dimensionality reduction method to extract effective features from an original dataset and improved the predictive capability of the ML model. Lian et al. [44] proposed an ML framework that combined empirical formulations and data-driven models; the results indicated that the use of prior knowledge can further improve the prediction performance of ML models. Peng et al. [45] determined that introducing physical constraints into an ML model can further improve the prediction performance and generalization capability of the model. It can be seen that model training strategies, such as reasonably preprocessing the features involved in training, can effectively improve prediction accuracy and reduce the impact of small data amounts on model prediction accuracy due to the difficulty in obtaining experimental data for ultrasonic rolling. Constrained by a small sample size, in order to improve the model’s prediction accuracy, data augmentation was used to reconstruct the original features. Additionally, the physical information during ultrasonic rolling is converted into features and added to the reconstructed dataset, completing the data preprocessing. The preprocessed dataset is then normalized and input into the ML model for training, ultimately using the model for predicting surface integrity. The overall process of ML model training is illustrated in Figure 3.

##### Feature Augmentation and Selection

Feature augmentation is a data processing method for raw datasets. Feature augmentation can transform an original dataset into a dataset that is suitable for ML models, thereby improving the ML model training when there is a small dataset. Feature augmentation includes a series of feature processing tasks, as shown in Figure 3. First, a feature dimension extension operation is performed on the original dataset to extend the feature dimensions of the dataset. Second, the feature importance weights of the extended dataset are calculated using a combination of the Shapley value (SHAP) method and the RF algorithm. Third, feature normalization is performed. Fourth, the features are added to the ML model in descending order according to importance weight. Then, the model performance is evaluated using the leave-one-out method to determine the optimal features for surface integrity prediction.

(1) Feature augmentation: During the ultrasonic rolling process, the surface integrity of a test bar was improved by adjusting the static pressure (P), the spindle speed (S), the feed rate (F), and the number of rolls (R). The initial surface roughness (Ra) of the test bar also affected the result of the ultrasonic rolling treatment; thus, it was also used as an input feature. However, with only these initial five input features, obtaining highly accurate prediction results was difficult, and the generalization capability of the trained ML model would be insufficient. The dimensionality of the input features significantly affected the prediction accuracy of the ML model, so it was necessary to expand the input feature dimensionality.

The ultrasonic rolling coverage rate was synergistically affected by the spindle speed and the feed rate, so a new initial feature, f, was constructed by multiplying the spindle speed and the feed rate.

Four functions, $x^{1/2}$, $x^{2}$, $x^{3}$, and ln(x + 1), for which x represents one of the six original features (i.e., P, S, F, R, Ra, and f), were used to generate 30 basic features [42]. Subsequently, these 30 basic features were multiplied pairwise, thereby producing 465 extended features (extended features numbers = 30+29+28+⋯+1). The basic and extended features were then merged, and duplicate features were removed. Ultimately, the initial six features were expanded to 456 features through the feature augmentation process.

It is not necessarily better to have more input features during ML model training. Beyond a critical point, more features could lead to the phenomenon known as the “curse of dimensionality”, in which the training performance of a model may worsen. Therefore, to quickly improve the training efficiency and to maximize the ability of the model to learn the complex nonlinear mapping relationship between the input features and the outputs, it was necessary to select the features with higher importance weights from the expanded 456 features and to determine the best features to use for model training.

**Figure 4:**
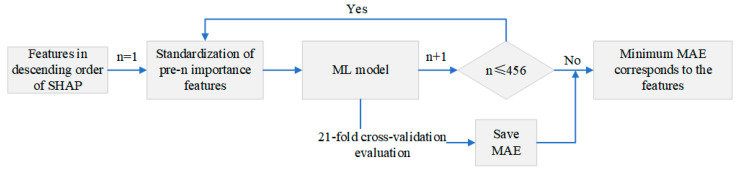
Flowchart of the feature selection process.

(2) Augmentation feature importance analysis: After dimensionality expansion was performed, the number of feature dimensions was 456. It was then necessary to extract appropriate features from these 456 features. After the features were sorted in descending order according to feature importance weight, they were input into the model for training to determine the optimal number of feature dimensions. The RF algorithm can capture the nonlinear relationships and interaction effects between complex features. Through the mean decrease in impurity method, the RF algorithm can comprehensively consider the individual contribution of each feature using multiple decision trees and can then evaluate the importance weights of the features. This method is widely used for feature importance analyses, but the RF algorithm calculation results are not applicable to models that are not tree-based, such as the SVM and GP models. To obtain feature importance results that apply to all model types, the SHAP method was also adopted to interpret the results of the RF algorithm calculations. The SHAP feature importance analysis method has the advantage of model independence. It can also provide a unified framework for feature importance analysis that can be used for different models. The 456 feature importance weights obtained during this study were ranked in descending order and were used to determine the best surface integrity prediction features.

(3) Feature selection method: It is important to ensure that feature selection is conducted without data leakage. When testing data are used for feature selection, there is a risk of fitting the model to the testing data, which compromises the ability to accurately evaluate the model’s performance on unseen data. Therefore, the feature selection process is performed entirely on the training dataset. The feature selection process is depicted in Figure 4. Based on the expanded 456 features sorted in descending order of SHAP importance, the pre-n importance features were selected for input into the ML algorithm. The assessment metric used was the mean absolute error (MAE), calculated by 21-fold cross-validation. The 456 features were sequentially input into the ML model, and all the MAE evaluation results were compared. The features corresponding to the smallest MAE values were determined to be the optimal surface integrity prediction features.

It should be noted that before model training could be performed, the features had to be standardized. The standardization caused all the features to have the same scale, though the original feature distribution was preserved, so deviations incurred by the different feature scales could be avoided to improve the accuracy of the model.

**Figure 5:**
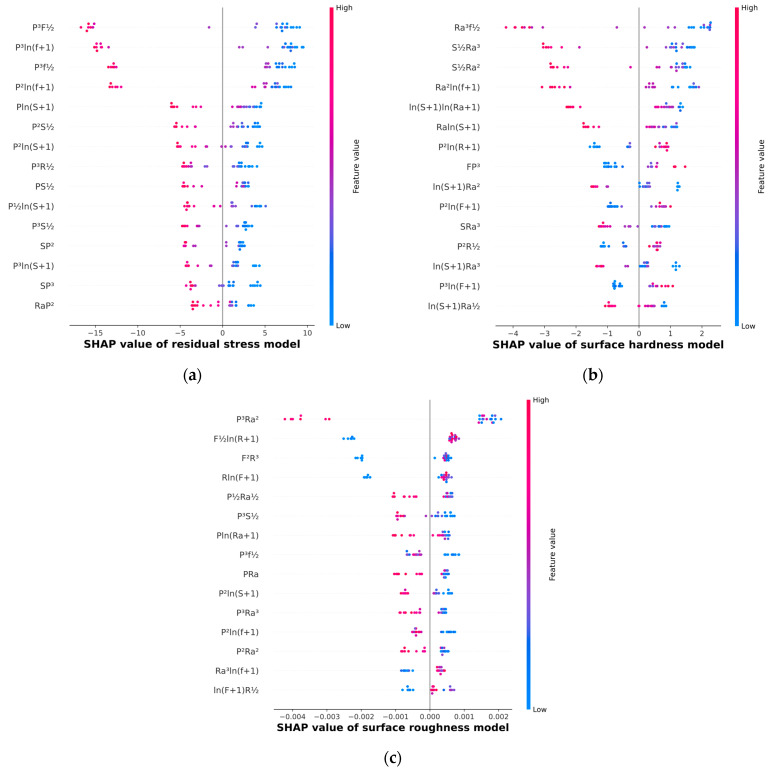
SHAP value analysis results: (**a**) residual stress results, (**b**) surface hardness results, and (**c**) surface roughness results.

(4) Feature selection results: After feature augmentation was performed, the SHAP method was used to conduct feature importance analyses for the surface roughness, surface hardness, and residual stress prediction tasks. The SHAP analysis results for feature importance are presented in Figure 5a–c. A larger deviation between the SHAP value and the baseline value indicates that the corresponding feature has greater importance. Figure 5a shows that static pressure (P) is included among the top five importance features, indicating that higher static pressure results in greater residual stress with other factors remaining constant. Similarly, Figure 5b shows that initial surface roughness (Ra) is included among the top five importance features, indicating that, with other factors remaining constant, higher initial surface roughness results in lower surface hardness. Figure 5c does not reveal any clear correlations. Table 2, Table 3 and Table 4 list the top 40 essential features in descending order according to importance weight along with their mean absolute SHAP values for the surface roughness, surface hardness, and residual stress prediction tasks, respectively.

**Figure 6:**
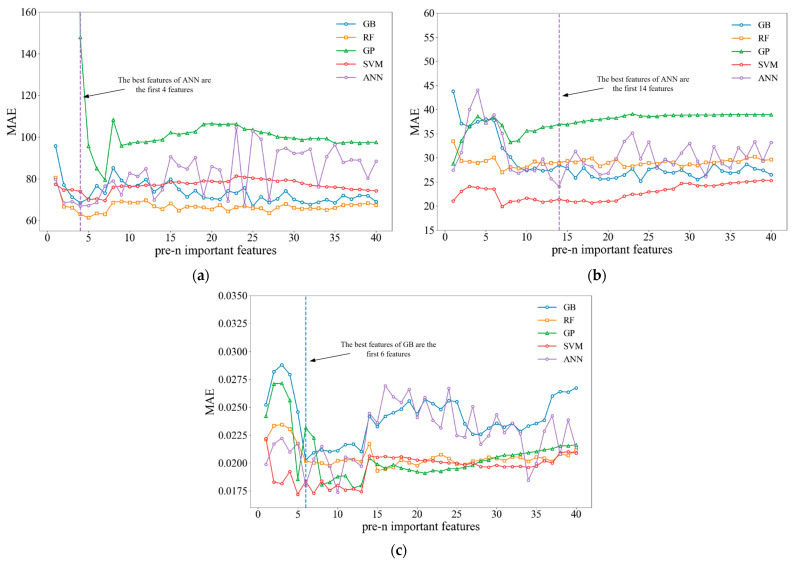
MAE values of the predictive models with different features: (**a**) residual stress predictions, (**b**) surface hardness predictions, and (**c**) surface roughness predictions.

Different ML algorithms have different principles and require different optimal input features to train surface integrity prediction models. In this study, three prediction tasks were investigated: surface roughness, surface hardness, and residual stress predictions. Five ML models were employed; these models had different default hyperparameters and required different numbers of input features. The MAE performance of each model was evaluated, and the results are presented in Figure 6.

As can be seen from the curves in Figure 6a–c, with the increase in input feature number, the MAE of ANN and GB fluctuates greatly. The MAE evaluation results for GP, SVM, and RF vary smoothly after reaching the minimum value, and the MAE value increases to varying degrees as the input features increase. The results for all three prediction tasks showed that once the optimal dimensionality was reached (i.e., the MAE reached its minimum value), further increasing the number of input features did not significantly improve the predictive accuracy of the ML model. Doing so may even produce a decrease in the predictive accuracy. The features corresponding to the smallest MAE values (features to the left of the dashed lines in Figure 6) represent the model training features that were ultimately used to predict the surface integrity, i.e., features were extracted in descending order according to the importance weight up to this position, after which the remaining features were discarded. The input features can be determined from Table 3, Table 4 and Table 5.

##### Physical Information

Theory-guided ML is an emerging paradigm that is focused on improving the learning capabilities of ML models using a priori theoretical knowledge. Because ultrasonic rolling experiments and data acquisition are associated with large workloads, model convergence must be accelerated, and prediction accuracies must be improved for small datasets. In this study, the physical information affiliated with the ultrasonic rolling process was compiled as input features to guide the model training.

**Figure 7:**
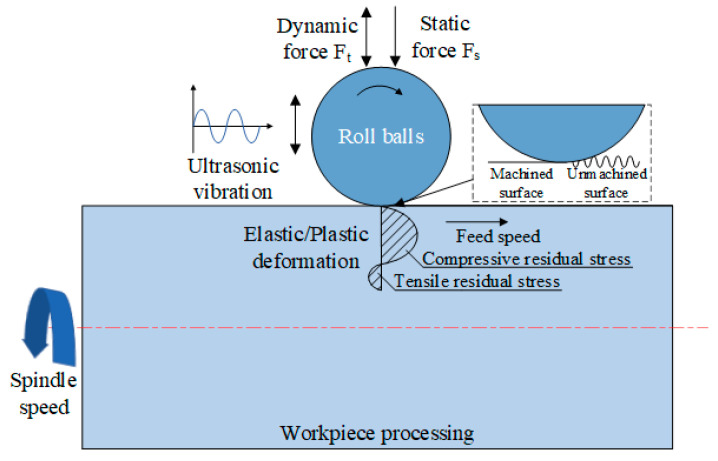
Schematic of the ultrasonic rolling process.

According to the Hertzian contact theory, the contact stress between the rolling ball and the test bar is critically influential to the plastic deformation degree experienced by the test bar surface and the surface quality improvement effect. An ultrasonic rolling force schematic is presented in Figure 7. Zheng et al. [17] calculated the maximum contact stress between the rolling ball and the test bar using the following equation:

(1) $$P_{max}=\sqrt[{3}]{\frac{6F_{d}E^{*2}}{\pi^{3}R^{*2}}}$$

where $F_{d}$ represents the equivalent impact force exerted by the rolling ball on the test bar; $E^{*}$ is the equivalent elastic modulus, which can be calculated using Equation (2); and $R^{*}$ is the equivalent radius, which can be obtained from Equation (3):

(2) $$\frac{1}{E^{*}}=\frac{1-v_{1}^{2}}{E_{1}}+\frac{1-v_{2}^{2}}{E_{2}}$$

(3) $$\frac{1}{R^{*}}=\frac{1}{R_{1}}+\frac{1}{R_{2}}$$

In Equation (2), $E_{1}$ and $E_{2}$ are the elastic moduli of the rolling ball and the test bar, respectively, $v_{1}$ and $v_{2}$ are the Poisson’s ratios for the rolling ball and the test bar, respectively. In Equation (3), $R_{1}$ and $R_{2}$ are the radii of the rolling ball and the test bar, respectively.

The impact force, F, consists of the static pressure, $F_{s}$, which the rolling ball exerts on the test bar, and the dynamic force, $F_{t}$, which is generated by the ultrasonic vibration of the rolling ball, as shown in the following equation:

(4) $$F=F_{s}+F_{t}$$

**Figure 8:**
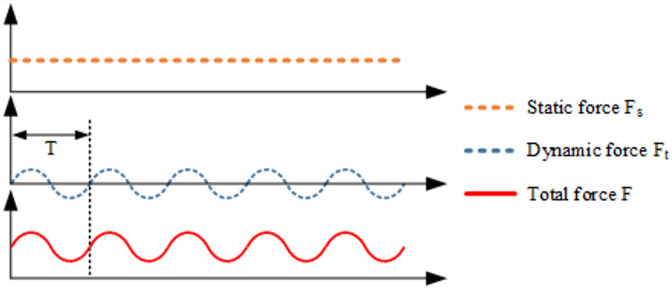
Relationships between the ultrasonic rolling static pressure, $F_{s}$, the dynamic force, $F_{t}$, and the total force, F.

The relationships between $F_{s}$, $F_{t}$, and F is presented in Figure 8. The vibration displacement of the rolling ball can be expressed by the following equation:

(5) $$y=A_{L}sin(2\pi ft)$$

where $A_{L}$ represents the amplitude of the rolling ball, and f is the vibration frequency of the rolling ball. The acceleration of the rolling ball can be expressed by the following equation:

(6) $$a=-A_{L}\omega^{2}sin(\omega t)$$

where ω=2πf. Therefore, the dynamic force, $F_{t}$, of the vibrating rolling ball can be obtained from the following equation:

(7) $$F_{t}=-A_{L}m\omega^{2}sin(\omega t)$$

where m represents the mass of the rolling ball. According to the impulse conservation theorem, the time-variant vibration force, F, of the rolling ball can be converted into an equivalent constant force, $F_{d}$. Within one cycle, $F_{d}$ can be expressed by the following equation:

(8) $$F_{d}=f\int_{0}^{\frac{1}{f}}Fdt$$

Therefore, for given ultrasonic frequency, ultrasonic amplitude, elastic modulus, Poisson’s ratio, rolling ball radius, and test bar radius values, the equivalent force of the rolling ball, $F_{d}$, can be calculated. Then, the maximum contact stress, $P_{max}$, can be obtained from Equation (1); this stress can then be used to characterize the physical information associated with the ultrasonic rolling process. The feature selection results were input into the ML model along with the physical information features to predict surface integrity.

#### 2.1.3. Model Training Strategy and Model Selection

Bayesian optimization is based on Bayes’ theorem, which dynamically adjusts the searching space for the next hyperparameter using previous observations. It achieves efficient optimization of the hyperparameters by performing iterations of this process. Bayesian optimization is widely used for optimizing model hyperparameters [46]. Therefore, before training the ML models, we use Bayesian optimization to determine the hyperparameters of each machine learning model to predict the three targets of surface residual stress, hardness, and surface roughness. Table 6 shows the search range of Bayesian optimization. The hyperparameters of each model for the three prediction tasks after Bayesian optimization was conducted are shown in Table 7, Table 8 and Table 9.

The ML models were trained with the training set, and the generalization capability and prediction accuracy of the models were evaluated with the test set. To evaluate the models’ performance, we use mean absolute error (MAE), mean absolute percentage error (MAPE), and root mean square error (RMSE) to characterize their predictive performance. MAPE calculation is shown in Equation (9), and RMSE calculation is shown in Equation (10):

(9) $$\mathrm{MAPE}=\frac{1}{n}\sum_{i=1}^{n}\left|\frac{Y_{i}-{\hat{Y}}_{i}}{Y_{i}}\right|\times 100\%$$

(10) $$\mathrm{RMSE}=\sqrt{\frac{1}{n}\sum_{i=1}^{n}{\left(Y_{i}-{\hat{Y}}_{i}\right)}^{2}}$$

where $Y_{i}$ represents the observed value of the ith sample, ${\hat{Y}}_{i}$ is the predicted value of the ith sample, and n is the total number of data samples. Small MAPE and RMSE values indicate minor errors in the model predictions.

**Figure 9:**
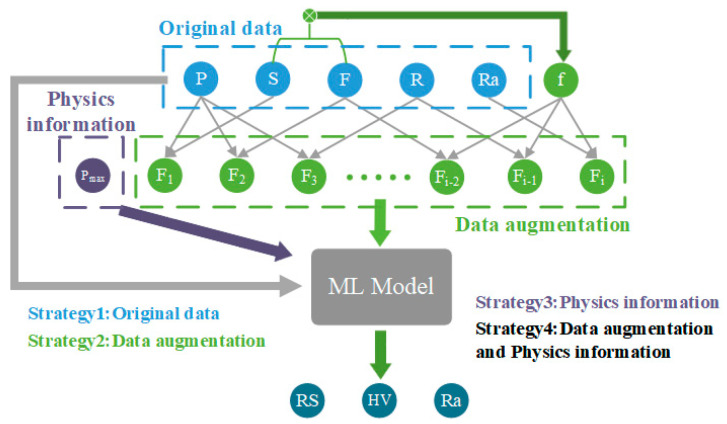
Illustration of the four strategies used to build the ML models.

This paper establishes artificial neural network (ANN), gradient boosting (GB), random forest (RF), Gaussian process (GP), and support vector machine (SVM) models using Python to predict the surface residual stress, hardness, and surface roughness after ultrasonic rolling. To compare the feature augmentation process and physical information effects on the performance improvements of the ML models, the model construction was divided into four strategies, as shown in Figure 9. In Strategy 1, the original dataset is used. Strategy 2 adds feature augmentation processing only, Strategy 3 only adds the physical information features to the original dataset, and Strategy 4 includes both the feature augmentation process and the physical information features.

### 2.2. Multi-Objective Optimization

The multi-objective optimization algorithm searches the solution set space to obtain a Pareto optimal solution set for three optimization objectives: residual stress, surface hardness, and surface roughness. In the context of multi-objective optimization, the Pareto set refers to the set of solutions that represent the optimal trade-offs among conflicting objectives. These solutions are not dominated by other solutions in the set, meaning no other solution offers improvements in one objective without worsening another. This paper adopts NSGA-II to efficiently search the solution space and obtain the Pareto front solutions, with the optimization goals of maximum RS (residual stress), maximum HV (surface hardness), and minimum Ra (surface roughness). The fitness values of RS, HV, and Ra in the NSGA-II optimization process were provided by the constructed ML model in Section 2.1. Therefore, the current multi-objective optimization problem is presented as below:

Maximize: RS(P,S,F,R,Ra)

Maximize:HV(P,S,F,R,Ra)

Minimize:Ra(P,S,F,R,Ra)

## 3. Results and Discussions

### 3.1. Residual Stress Predictions and Model Evaluation

**Figure 10:**
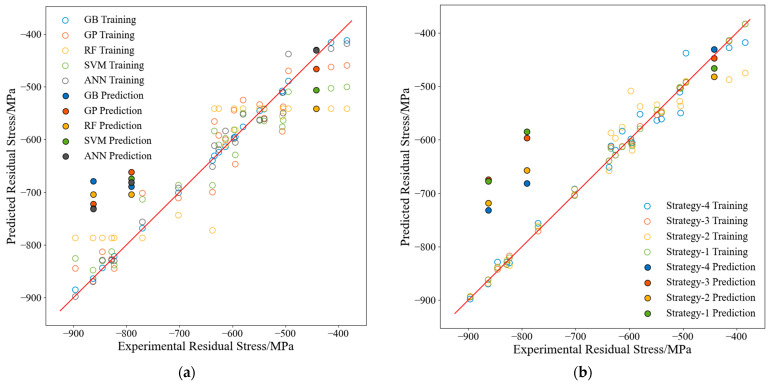
Performance of the residual stress prediction model: (**a**) comparison of all models, (**b**) comparison of the ANN models built with different strategies.

ML models were constructed to predict the residual stresses using feature augmentation and physical information features (Strategy 4). A comparison between the prediction results from each ML model and the actual experimental results is presented in Figure 10a. Table 10 provides the evaluation results for each ML model included in Figure 10a in terms of the MAE, MAPE, and RMSE metrics. Analyses of Figure 10a and Table 10 indicated that the ANN model performed best in terms of the MAE, MAPE, and RMSE evaluation metrics. This is because ANNs are universal approximators capable of mapping complex nonlinear functions [38]. The training features used for the ANN model were the first four features shown in Figure 6a and Table 3.

To analyze the feature augmentation and physical information effects on the ANN model prediction performance, the prediction results of the four strategies were compared with the actual results in Figure 10b. Table 11 presents the prediction performance metrics of the ANN models for each of the four strategies. Figure 10b and Table 11 show that the prediction results of the models built using Strategy 4 performed significantly better than the models built using Strategy 1. The models built using Strategy 3 had a slightly improved predictive ability; the models built using Strategy 2 also had an improved predictive ability; and adding the physical information features to the feature augmentation (Strategy 2) could further improve the predictive performance. Taking the ANN model as an example, the MAE values obtained from Strategies 1, 2, 3, and 4 were 138.56, 106.14, 129.21, and 84.20, respectively; the MAPE values were 17.66%, 14.24%, 15.83%, and 10.58%, respectively; and the RMSE values were 160.75, 116.06, 156.35, and 98.91, respectively. These results show that combining the feature augmentation with the physical information features effectively improved the accuracy and generalization capability of the residual stress prediction model.

### 3.2. Surface Hardness Predictions and Model Evaluation

**Figure 11:**
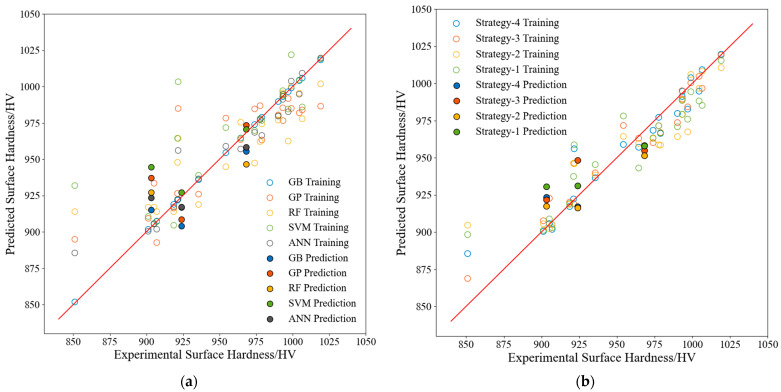
Performance of the surface hardness prediction model: (**a**) comparison of all models, (**b**) comparison of the ANN models built with different strategies.

Next, the surface hardness after ultrasonic rolling was predicted using models built using Strategy 4, and a comparison between the prediction results of each model and the actual values is presented in Figure 11a. The prediction performance evaluation metrics for each ML model compared in Figure 11a are listed in Table 12. Analyses of the results shown in Figure 11a and Table 12 indicated that the ANN model performed best with respect to all three evaluation metrics. The best training features for the ANN model were the first 14 features, as shown in Figure 6b and Table 4. In addition, the predictive performance of each model on the test set is good because the surface hardness data in the dataset are clustered in the range of 900–1000 HV. Therefore, if more samples are used for model training, the prediction accuracy in this range will be higher.

Comparisons of the prediction results obtained from the ANN models built using each of the four strategies are illustrated in Figure 11b and are listed in Table 13. The models built using both feature augmentation and physical information features (Strategy 4) surpassed the models built using the other three strategies. The results further demonstrated that including both feature augmentation and physical information features improved the predictive performance and generalization capabilities of the models.

### 3.3. Surface Roughness Predictions and Model Evaluation

**Figure 12:**
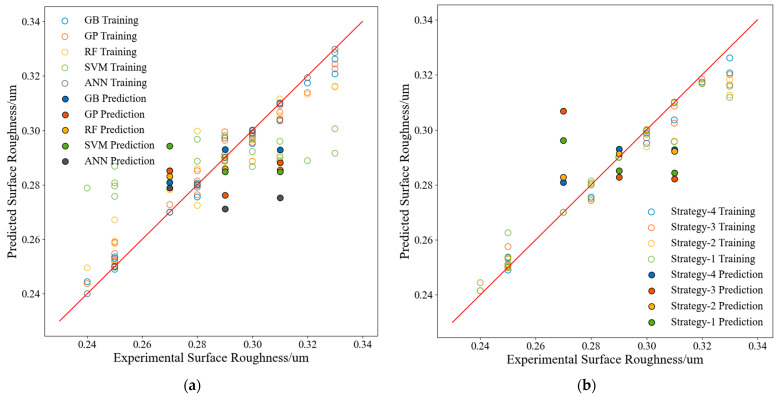
Performance of the surface roughness prediction model: (**a**) comparison of all models, (**b**) comparison of the GB models built with different strategies.

Strategy 4 was also used to construct models to predict the surface roughness. A comparison between the prediction results of these models and the actual values is presented in Figure 12a, and the performance evaluation metrics of each model are listed in Table 14. Figure 12a shows that the GB model performed best for the surface roughness prediction task. The best features of the GB model were the first six features shown in Figure 6c and Table 5.

Figure 12b, along with Table 15, compares the prediction performance characteristics of the GB models constructed with the four strategies. These comparisons verify again that the best prediction performance was achieved by models constructed using both feature augmentation and physical information features.

### 3.4. Optimization and Validation of Process Parameters

Section 2.2 determines the optimization objectives as maximum RS, maximum HV, and minimum Ra. The RS, HV, and Ra prediction models established above are used as objective functions. The NSGA-II algorithm is employed to search the solution space to find the Pareto optimal solution. The search space is based on the experimental scheme in Table 1, as shown in the following equations:

Subject to:

- 300≤P≤1000 step: 25 N,
- 50≤S≤300 step: 25 rpm,
- 0.06≤F≤0.36 step: 0.01 mm/r,
- 1≤R≤6 step: 1 round.

This ensures that the response of the entire process parameter search space can be predicted by the established ML model. Additionally, different step sizes are set for each parameter to constrain the searched process parameters to be directly applicable for processing, preventing the identification of impractical process parameters. The NSGA-II algorithm is configured with a population size of 20 and a genetic generation of 900 iterations. The NSGA-II search process adopts an elite strategy to ensure that the optimal individuals are retained for the next iteration, thus speeding up the search for the solution space.

**Figure 13:**
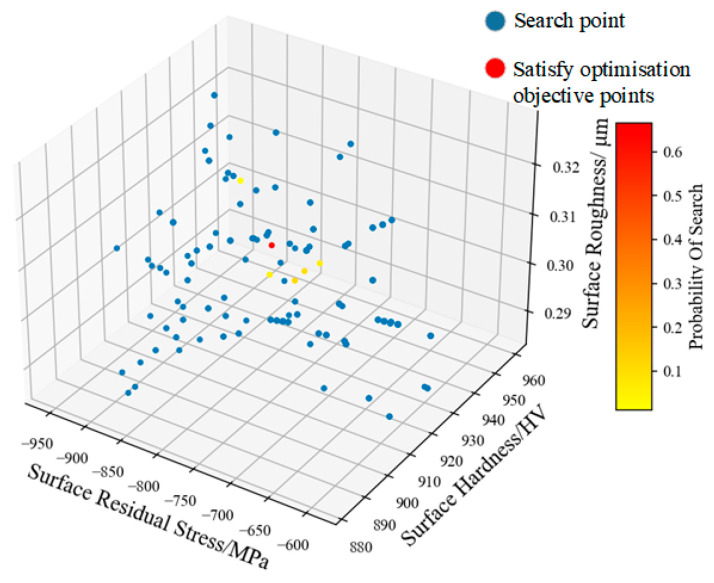
The Pareto front solutions obtained by the NSGA-II search.

The NSGA-II algorithm was run five times to test the robustness of the proposed method, and the probability of each set of process parameters appearing in the Pareto rank 1 solution set from the final iteration was recorded. The results are shown in Table 16, and Figure 13. As shown in Table 16, when the initial surface roughness of the test bar is 0.54 µm, the maximum probability of occurrence process parameters are a static pressure of 900 N, a spindle speed of 75 rpm, a feed rate of 0.19 mm/r, and rolling once, which meets the economic requirement of one roll.

Ultrasonic rolling tests are conducted using the optimized parameters to verify the effectiveness of the optimized process parameters, and the residual stress, surface hardness, and surface roughness after rolling are measured. The surface roughness of the test bar after ultrasonic rolling with the optimized parameters was reduced to 0.32 µm, the surface hardness reached 958.23 HV, and the surface residual stress reached −920.60 MPa.

The optimized residual stress reached a maximum compared to the training dataset. At the same time, surface hardness and surface roughness showed significant improvements but did not reach the optimal levels compared to the training dataset. This is because the initial surface roughness of the test bar used for parameter optimization was relatively large, reaching 0.54 µm. From Figure 5b, it can be observed that the six most important features affecting surface hardness all include the initial surface roughness, and they all have the characteristic that larger feature values correspond to smaller SHAP values. This indicates that an increase in initial surface roughness negatively contributes to surface hardness. Regarding surface roughness, the degree of improvement is the largest compared to the other process parameters. The ML combined with NSGA-II proposed in this paper has effectively optimized the process parameters to achieve maximum residual stress and surface hardness while minimizing surface roughness. The proposed method is effective.

**Figure 14:**
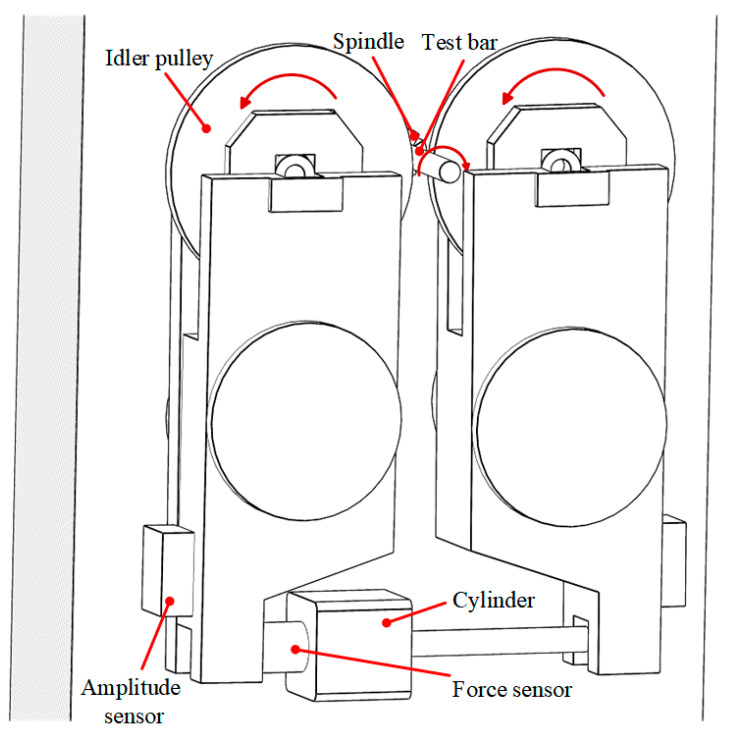
Rolling contact fatigue test machine.

In addition, the contact fatigue test machine shown in Figure 14 was used to test the fatigue performance of an untreated bar, a bar without parameter optimization, and a bar with optimized parameters. The air spindle is used to drive the test bar to rotate. The two idler wheels in the figure are driven wheels. The contact stress exerted on the test bar by the idler is 5 GPa, and the test machine’s operation and the bar’s fatigue failure are monitored using an amplitude sensor. Without parameter optimization, three bars are selected from Table 1. Bar 1 has a static pressure of 900 N, a spindle speed of 200 rpm, a feed rate of 0.06 mm/r, and six rolling passes. Bar 2 has a static pressure of 800 N, a spindle speed of 200 rpm, a feed rate of 0.24 mm/r, and one rolling pass. Bar 3 has a static pressure of 700 N, a spindle speed of 50 rpm, a feed rate of 0.24 mm/r, and three rolling passes.

The test results showed that the contact fatigue life of the untreated bar was 1.98 × 10^7^ cycles, the contact fatigue life of bar 1 without parameter optimization was 2.73 × 10^7^ cycles, the contact fatigue life of bar 2 without parameter optimization was 2.64 × 10^7^, the contact fatigue life of bar 3 without parameter optimization was 2.36 × 10^7^, and the contact fatigue life of the bar with optimized parameters was 3.02× 10^7^. After optimization, the bar’s contact fatigue life was 52.5% higher than that of the untreated bar, 10.6% higher than that of bar 1 without parameter optimization, 14.4% higher than that of bar 2 without parameter optimization, and 28.0% higher than that of bar 3 without parameter optimization. Therefore, it can be concluded that the process parameter optimization method proposed in this paper can effectively find the optimal process parameters based on the optimization objectives and further improve the contact fatigue life.

## 4. Conclusions

This paper proposes a method for designing ultrasonic rolling process parameters using a machine learning model combined with a multi-objective optimization algorithm. By feature augmentation and constructing physical information features, the prediction performance of the ML model on small-sample datasets is improved. This method links ultrasonic rolling process parameters with the surface integrity (surface roughness, surface hardness, residual stress) after rolling. Subsequently, the NSGA-II is used to optimize the ultrasonic rolling process parameters. This approach can improve the efficiency of selecting ultrasonic rolling process parameters and reduce experimental workload. The conclusions drawn in this paper are as follows:

1. Experimental tests of ultrasonic rolling were conducted according to a uniform design experimental scheme, and a dataset was established. The optimal features for predicting surface residual stress, surface hardness, and surface roughness were obtained through feature augmentation. Additionally, physical information features were constructed and input into the machine learning (ML) model for prediction. By comparing the prediction performance of five machine learning models (ANN, GB, RF, GP, and SVM) on the three prediction tasks, the best-performing ML model was selected for each prediction task. Furthermore, the prediction performance of four strategies (original data only, feature augmentation only, physical information only, and feature augmentation combined with physical information) was compared. The results showed that the feature augmentation and physical information-guided ML models exhibited the best prediction performance. This demonstrates that feature augmentation and physical information guidance can effectively improve the model’s generalization ability and prediction accuracy on small-sample datasets.
2. Based on comparing the prediction performance of various models on the three prediction tasks, an ANN model was established to predict surface residual stress and surface hardness, while a GB model was established to predict surface roughness. The NSGA-II was employed to rapidly search for the optimal process parameters, simultaneously maximizing surface residual stress and hardness while minimizing surface roughness. The optimization was performed on a test bar with an initial surface roughness of 0.54 µm, and the optimized process parameters were a static pressure of 900 N, a spindle speed of 75 rpm, a feed rate of 0.19 mm/r, and rolling once.
3. With the optimization process parameters for ultrasonic rolling, the surface residual stress was −920.60 MPa, the surface hardness reached 958.23 HV, and the surface roughness was reduced to 0.32 µm. The optimized residual stress was maximized, and the reduction in surface roughness was the greatest compared to the training dataset. The improvement in surface hardness did not reach the optimal level due to the large initial surface roughness, which had a negative contribution to the increase in hardness. Additionally, the contact fatigue test results showed that the fatigue life of the bar with the optimized process parameters reached 3.02 × 10^7^ cycles, which was 10.6% higher than that of bar 1 with the unoptimized process parameters, 14.4% higher than that of bar 2 without parameter optimization, 28.0% higher than that of bar 3 without parameter optimization, and 52.5% higher than that of the untreated bar. These results validate the feasibility and effectiveness of this study’s process parameter optimization method.

The ML combined with the NSGA-II approach proposed in this study can provide an advanced tool for the optimization of ultrasonic rolling process parameters. However, there are some limitations in this study. In addition to the process parameters static pressure, spindle speed, feed rate, and number of rolls, there are other parameters in the ultrasonic rolling process that do not take into account the effects of different level values, such as amplitude, frequency, rolling ball diameter, etc. We will continue to increase the characteristics of the original parameters in order to further improve the prediction performance of the model according to the method proposed in this paper. In addition, we can further investigate the influence mechanism of surface residual stress, surface hardness, and surface roughness on the contact fatigue life, and clarify the optimization objective of the optimal contact fatigue life, in order to find the parameters of the ultrasonic rolling process when the fatigue life is optimal. Finally, different multi-objective optimization algorithms may have different effects on the results, which is also a very important research direction. We will consider conducting detailed comparisons of different multi-objective evolutionary algorithms and investigating their effects on the optimization results in subsequent research.

## Figures and Tables

**Table 1:** Experimental results of different process parameters.

No.	P	S	F	R	Initial Ra	RS	HV	Rolled Ra
1	600	250	0.06	1	0.28	−625.80	977.79	0.25
2	500	50	0.06	4	0.33	−597.00	993.48	0.31
3	700	50	0.24	3	0.38	−505.91	993.28	0.29
4	500	150	0.36	1	0.39	−579.90	924.03	0.32
5	800	200	0.24	1	0.33	−845.47	952.33	0.28
6	400	250	0.12	5	0.37	−442.20	907.05	0.31
7	800	300	0.18	4	0.31	−823.21	974.03	0.31
8	400	100	0.12	2	0.32	−494.31	921.28	0.27
9	500	200	0.18	3	0.35	−548.60	903.24	0.33
10	900	200	0.06	6	0.30	−896.50	921.69	0.25
11	800	150	0.06	3	0.35	−701.93	997.00	0.24
12	700	200	0.36	4	0.37	−769.95	935.66	0.29
13	600	250	0.30	5	0.30	−613.10	964.49	0.30
14	900	150	0.24	4	0.25	−863.10	999.09	0.30
15	400	100	0.30	5	0.45	−413.90	918.69	0.33
16	400	250	0.30	2	0.31	−383.90	851.01	0.28
17	500	300	0.24	6	0.36	−504.30	901.23	0.29
18	600	100	0.30	2	0.35	−540.00	978.94	0.29
19	700	150	0.18	6	0.36	−595.58	968.15	0.28
20	900	50	0.18	1	0.33	−862.60	1019.18	0.25
21	700	300	0.12	2	0.40	−637.69	905.22	0.25
22	600	100	0.12	5	0.36	−634.00	990.19	0.31
23	900	300	0.36	3	0.28	−828.06	1006.57	0.27
24	800	50	0.36	6	0.38	−791.07	1004.54	0.30

**Table 2:** Statistical analysis and distribution test of the train: test dataset.

Data Split	N No.	Mean	Stdev	Min	Q1	Med	Q3	Max	IQR	Skew	Kur	KS Test
CS_Train	21	−638.16	146.14	−896.5	−769.95	−613.10	−540.00	−383.9	229.95	−0.24	−0.95	0.63
CS_Test	3	−698.62	183.66	−862.6	−826.84	−791.07	−616.64	−442.2	210.20	0.63	−1.5	
CS_dataset	24	−645.72	152.66	−896.5	−799.11	−619.45	−531.48	−383.9	267.63	−0.12	−1.19	
HV_Train	21	957.84	44.14	851.01	921.28	974.03	993.48	1019.18	72.20	−0.62	−0.55	0.39
HV_Test	3	931.81	27.06	903.24	913.64	924.03	946.09	968.15	32.45	0.41	−1.50	
HV_dataset	24	954.59	43.25	851.01	920.63	966.32	993.33	1019.18	72.70	−0.45	−0.72	
Ra_Train	21	0.29	0.027	0.24	0.27	0.29	0.31	0.33	0.04	−0.20	−1.00	0.99
Ra_Test	3	0.29	0.016	0.27	0.28	0.29	0.30	0.31	0.02	0	−1.50	
Ra_dataset	24	0.29	0.026	0.24	0.27	0.29	0.31	0.33	0.04	−0.22	−0.90	

**Table 3:** Most influential features for the surface residual stress predictions and the corresponding SHAP values.

No.	Feature	SHAP Value	No.	Feature	SHAP Value
1	P^3^F^1/2^	9.68	11	P^3^S^1/2^	2.96
2	P^3^ln(f + 1)	9.64	12	SP^2^	2.67
3	P^3^f^1/2^	8.19	13	P^3^ln(S + 1)	2.62
4	P^2^ln(f + 1)	7.68	14	SP^3^	2.50
5	Pln(S + 1)	3.60	15	P^2^Ra	2.16
6	P^2^S^1/2^	3.44	16	P^2^ln(P + 1)	1.98
7	P^2^ln(S + 1)	3.15	17	P^4^	1.93
8	P^3^R^1/2^	3.04	18	P^3^Ra	1.91
9	PS^1/2^	3.04	19	P^3^ln(R + 1)	1.83
10	P^1/2^ln(S + 1)	2.99	20	P^3^ln(Ra + 1)	1.80

**Table 4:** Most influential features for the surface hardness predictions and the corresponding SHAP values.

No.	Feature	SHAP Value	No.	Feature	SHAP Value
1	Ra^3^f^1/2^	2.37	11	Ra^3^S	0.77
2	Ra^3^S^1/2^	1.69	12	P^2^R^1/2^	0.70
3	Ra^2^S^1/2^	1.60	13	Ra^3^ln(S + 1)	0.68
4	Ra^2^ln(f + 1)	1.53	14	P^3^ln(F + 1)	0.66
5	ln(S + 1)ln(Ra + 1)	1.32	15	Ra^1/2^ln(S + 1)	0.61
6	Raln(S + 1)	1.00	16	P^3^R^1/2^	0.49
7	P^2^ln(R + 1)	0.86	17	P^2^F^1/2^	0.43
8	FP^3^	0.82	18	P^3^ln(R + 1)	0.42
9	ln(S + 1)Ra^2^	0.79	19	P^3^F^1/2^	0.35
10	P^2^ln(F + 1)	0.78	20	FP^2^	0.33

**Table 5:** Most influential features for the surface roughness predictions and the corresponding SHAP values.

No.	Feature	SHAP Value	No.	Feature	SHAP Value
1	P^3^Ra^2^	0.00226	11	P^3^Ra^3^	0.00045
2	F^1/2^ln(R + 1)	0.00109	12	P^2^ln(f + 1)	0.00045
3	F^2^R^3^	0.00086	13	P^2^Ra^2^	0.00044
4	Rln(F + 1)	0.00080	14	Ra^3^ln(f + 1)	0.00043
5	P^1/2^Ra^1/2^	0.00064	15	ln(F + 1)R^1/2^	0.00037
6	P^3^S^1/2^	0.00056	16	RF^2^	0.00036
7	Pln(Ra + 1)	0.00055	17	P^2^ln(Ra + 1)	0.00033
8	P^3^f^1/2^	0.00052	18	RF^3^	0.00030
9	PRa	0.00051	19	ln(F + 1)ln(R + 1)	0.00029
10	P^2^ln(S + 1)	0.00047	20	FR^1/2^	0.00029

**Table 6:** The search range of Bayesian optimization.

Model	Searched Hyperparameters
GB	Number of estimators: {100, 200, 300, 400, 500}, Maximum depth: {3, 4, 5} Minimum samples leaf: {1, 2, 3}, Minimum samples split: {2, 3, 4} Learning rate: {0.01, 0.1, 0.2} Loss function: {huber, absolute error, squared error}
ANN	Hidden layer sizes: {(100), (100, 50), (100, 50, 10)}, Solver: {adam} Activation function: {rule, tanh}, Learning rate: {0.001, 0.01, 0.1} Maximum number of iterations: {50, 200, 300}, Batch size: {6, 12, 21}
GP	Kernel: {RBF, RBF + WhiteKernel}, Alpha: {1 × ^10−5^, 1 × 10^−4^, 1 × 10^−3^, 1 × 10^−2^} Number of restarts optimizer: {0, 3, 5, 8, 10}
RF	Number of estimators: {300, 400, 500}, Maximum depth: {5, 10, 20, 30, 50} Minimum samples leaf: {2, 8, 10}, Minimum samples split: {1, 2, 5, 10}
SVM	Kernel: {rbf, linear, poly}, C: {0.01, 0.1, 1.0, 10, 100} Gamma: {scale, 0.001, 0.01, 0.1, 1}, Epsilon: {0.001, 0.01, 0.1, 1, 10, 100}

**Table 7:** The hyperparameters for training the residual stress prediction model.

Model	Optimal Hyperparameters
GB	Number of estimators: 500, Maximum depth: 3 Minimum samples leaf: 2, Minimum samples split: 3 Learning rate: 0.01, Loss function: huber
ANN	Hidden layer sizes: (100, 50), Activation function: rule Solver: adam, Maximum number of iterations: 200 Learning rate: 0.001, Batch size: 21
GP	Kernel: RBF + WhiteKernel, Alpha: 1 × 10^−5^ Number of restarts optimizer: 10
RF	Number of estimators: 500, Maximum depth: 5 Minimum samples leaf: 8, Minimum samples split: 2
SVM	Kernel: rbf, C: 1.0 Gamma: scale, Epsilon: 0.1

**Table 8:** The hyperparameters for training the surface hardness prediction model.

Model	Optimal Hyperparameters
GB	Number of estimators: 500, Maximum depth: 4 Minimum samples leaf: 1, Minimum samples split: 2 Learning rate: 0.01, Loss function: squared error
ANN	Hidden layer sizes: (100, 50, 10), Activation function: rule Solver: adam, Maximum number of iterations: 200 Learning rate: 0.001, Batch size: 12
GP	Kernel: RBF + WhiteKernel, Alpha: 0.001 Number of restarts optimizer: 10
RF	Number of estimators: 500, Maximum depth: 50 Minimum samples leaf: 2, Minimum samples split: 10
SVM	Kernel: poly, C: 100.0 Gamma: 0.01, Epsilon: 0.001

**Table 9:** The hyperparameters for training the surface roughness prediction model.

Model	Optimal Hyperparameters
GB	Number of estimators: 400, Maximum depth: 4 Minimum samples leaf: 2, Minimum samples split: 4 Learning rate: 0.01, Loss function: absolute error
ANN	Hidden layer sizes: (100, 50), Activation function: relu Solver: adam, Maximum number of iterations: 200 Learning rate: 0.001, Batch size: 6
GP	Kernel: RBF + WhiteKernel, Alpha: 0.01 Number of restarts optimizer: 3
RF	Number of estimators: 500, Maximum depth: 30 Minimum samples leaf: 2, Minimum samples split: 2
SVM	Kernel: linear, C: 0.01 Gamma: 0.001, Epsilon: 0.001

**Table 10:** Performance metrics of all model types for the residual stress prediction task.

ML Model	MAE	MAPE	RMSE
ANN	84.20	10.58%	98.91
GB	99.13	12.29%	121.38
RF	114.78	17.26%	118.94
GP	97.68	12.66%	110.74
SVM	104.40	14.87%	108.35

**Table 11:** Performance metrics for the ANN models built using the four strategies for the residual stress prediction task.

ML Model	Category	MAE	MAPE	RMSE
ANN	Strategy 1	138.56	17.66%	160.75
	Strategy 2	106.14	14.24%	116.06
	Strategy 3	129.21	15.83%	156.35
	Strategy 4	84.20	10.58%	98.91

**Table 12:** Performance metrics of all model types for the surface hardness prediction task.

ML Model	MAE	MAPE	RMSE
ANN	12.34	1.34%	13.60
GB	14.90	1.60%	15.32
RF	17.54	1.88%	19.05
GP	18.27	2.00%	21.77
SVM	15.57	1.72%	23.93

**Table 13:** Performance metrics for the ANN models built using the four strategies for the surface hardness prediction task.

ML Model	Category	MAE	MAPE	RMSE
ANN	Strategy 1	14.87	1.62%	17.34
	Strategy 2	12.93	1.38%	13.47
	Strategy 3	18.70	2.02%	19.23
	Strategy 4	12.34	1.34%	13.35

**Table 14:** Performance metrics of all model types for the surface roughness prediction task.

ML Model	MAE	MAPE	RMSE
ANN	0.0208	6.99%	0.0234
GB	0.0104	3.55%	0.0119
RF	0.0139	4.72%	0.0162
GP	0.0170	5.82%	0.0173
SVM	0.0182	6.28%	0.0204

**Table 15:** Performance metrics for the GB models built using the four strategies.

ML Model	Category	MAE	MAPE	RMSE
ANN	Strategy 1	0.0189	6.53%	0.0213
	Strategy 2	0.0106	3.63%	0.0127
	Strategy 3	0.0240	8.37%	0.0270
	Strategy 4	0.0104	3.55%	0.0119

**Table 16:** Statistical results of process parameter searches.

No.	P	S	F	R	Probability
1	900	75	0.19	1	66.6%
2	900	50	0.17	1	11.5%
3	925	100	0.20	1	10.3%
4	875	150	0.11	1	5.7%
5	925	75	0.1	2	4.6%
6	950	100	0.12	3	1.1%

## Data Availability

Dataset available on request from the authors.
